# Recurrent pulmonary vein stenosis in a pediatric bilateral lung transplant recipient

**DOI:** 10.1016/j.jhlto.2026.100650

**Published:** 2026-07-29

**Authors:** Timothy Klouda, Francis Fynn-Thompson, Kathy Jenkins, Michael Farias, Christina M. Ireland, Jennifer Mosey, Wai Wong, Saba Sheikh, Christian Benden, Gary Visner

**Affiliations:** a Division of Pulmonary Medicine, Boston Children's Hospital, Harvard Medical School, Boston, MA; b Department of Cardiac Surgery, Boston Children's Hospital, Harvard Medical School, Boston, MA; c Department of Cardiology, Boston Children's Hospital; Harvard Medical School, Boston, MA

**Keywords:** pediatric, lung transplant, pulmonary vein stenosis, pulmonary vascular disease

## Abstract

Pulmonary vein stenosis (PVS) is characterized by luminal narrowing from neointimal proliferation, leading to the obstruction of pulmonary venous blood flow. Lung transplantation is considered a definitive treatment for patients with severe and progressive PVS that is unresponsive to treatment. We present a case in which a three-year-old female who received a bilateral lung transplant for PVS was diagnosed with disease recurrence in multiple veins. This case highlights the need to discuss the possibility of PVS returning during pre-transplant counseling and advocates for frequent monitoring in the post-transplant period.

## Introduction

Pulmonary vein stenosis (PVS) is characterized by luminal narrowing from intimal proliferation and fibrosis, leading to obstruction of blood flow and pulmonary hypertension (PH). While patient outcomes have improved with multimodal therapy, lung transplantation is recommended for refractory cases. PVS can occur after transplantation due to technical complications; however, the recurrence of primary disease has not been reported. We present a case of recurrent intraluminal PVS diagnosed after lung transplantation in a pediatric patient, emphasizing the need for monitoring and appropriate counseling when offering transplantation as a definitive therapy for PVS.

## Case presentation

**Figure 1:**
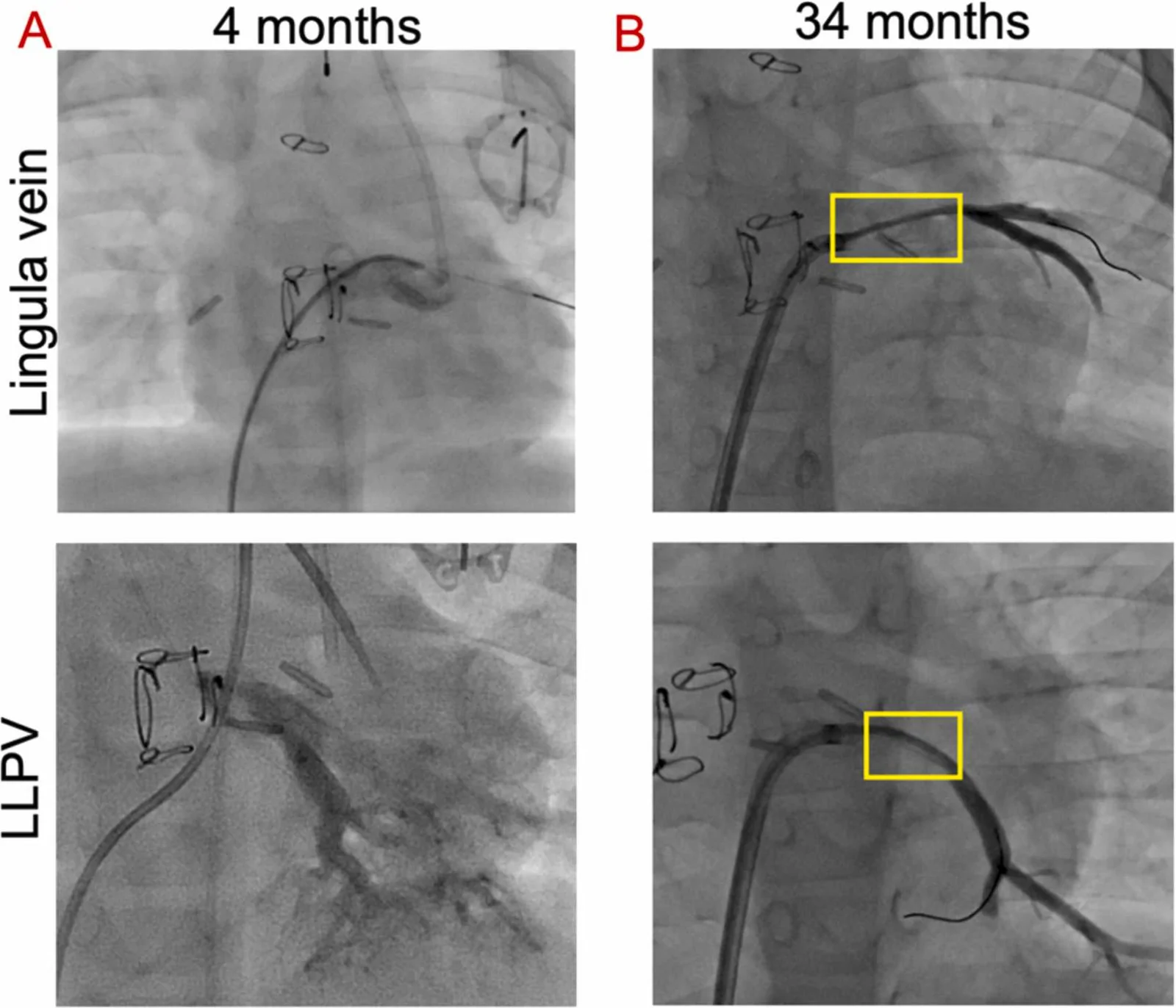
Cardiac catheterization reveals recurrent left-sided intraluminal PVS.(A) Angiography four months after transplantation showing a patent lingual vein and LLPV. (B) Angiography 34 months after transplantation, showing severe stenosis of the lingular vein and LLPV highlighted with yellow boxes.

**Figure 2:**
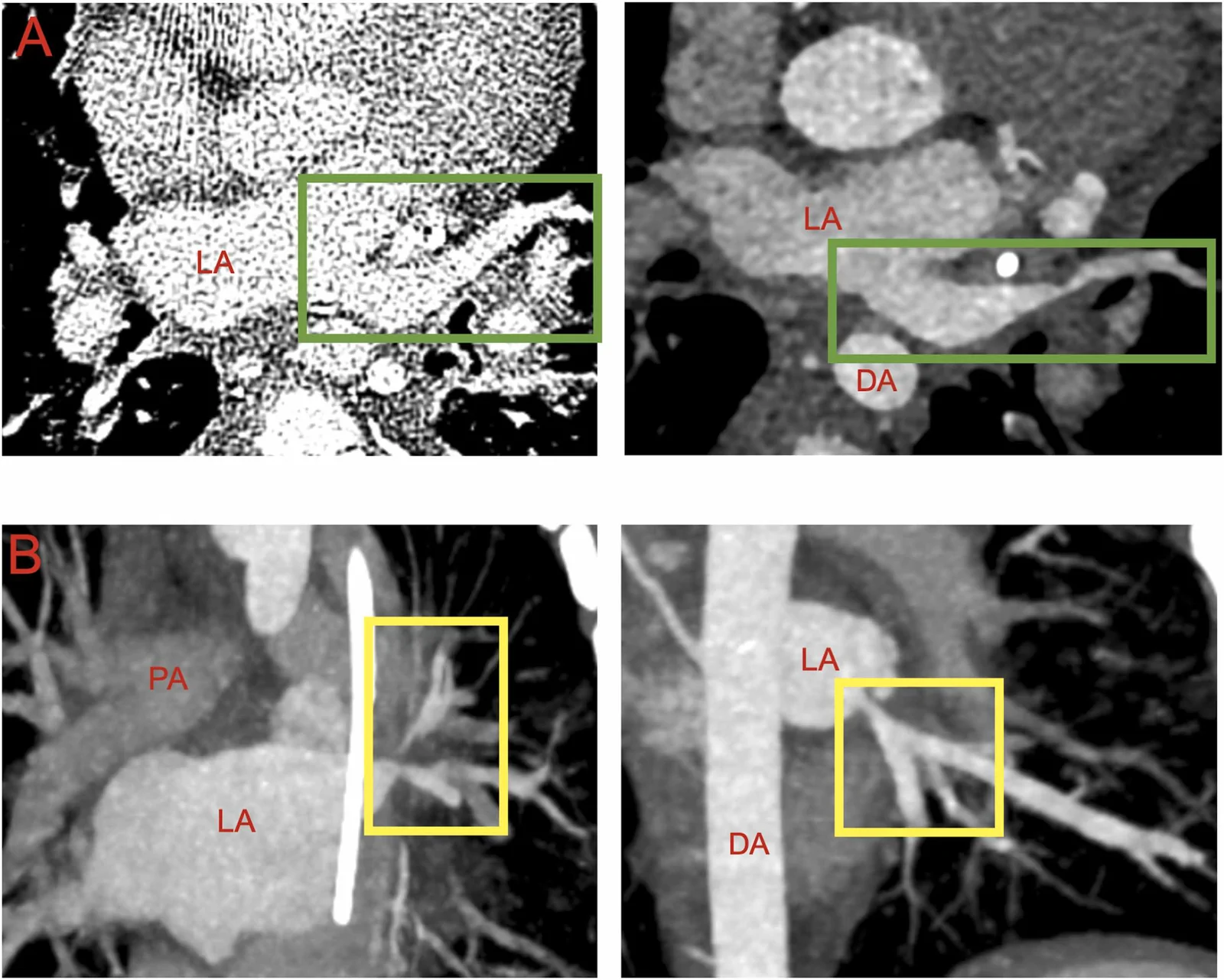
Chest CT findings of recurrent PVS post lung transplantation. (A) Axial views of the Chest CT obtained 11 months after transplant (left) and cardiac CT 34 months after transplantation (right). Note the distal narrowing of the left pulmonary vein highlighted in the green box. (B) Coronal views of the cardiac CT obtained 34 months after transplant, demonstrating stenosis (yellow boxes) of the left upper vein (left) and atresia/stenosis of the left lower vein (right). *DA: descending aorta, LA: left atrium, PA: pulmonary artery.*

**Figure 3:**
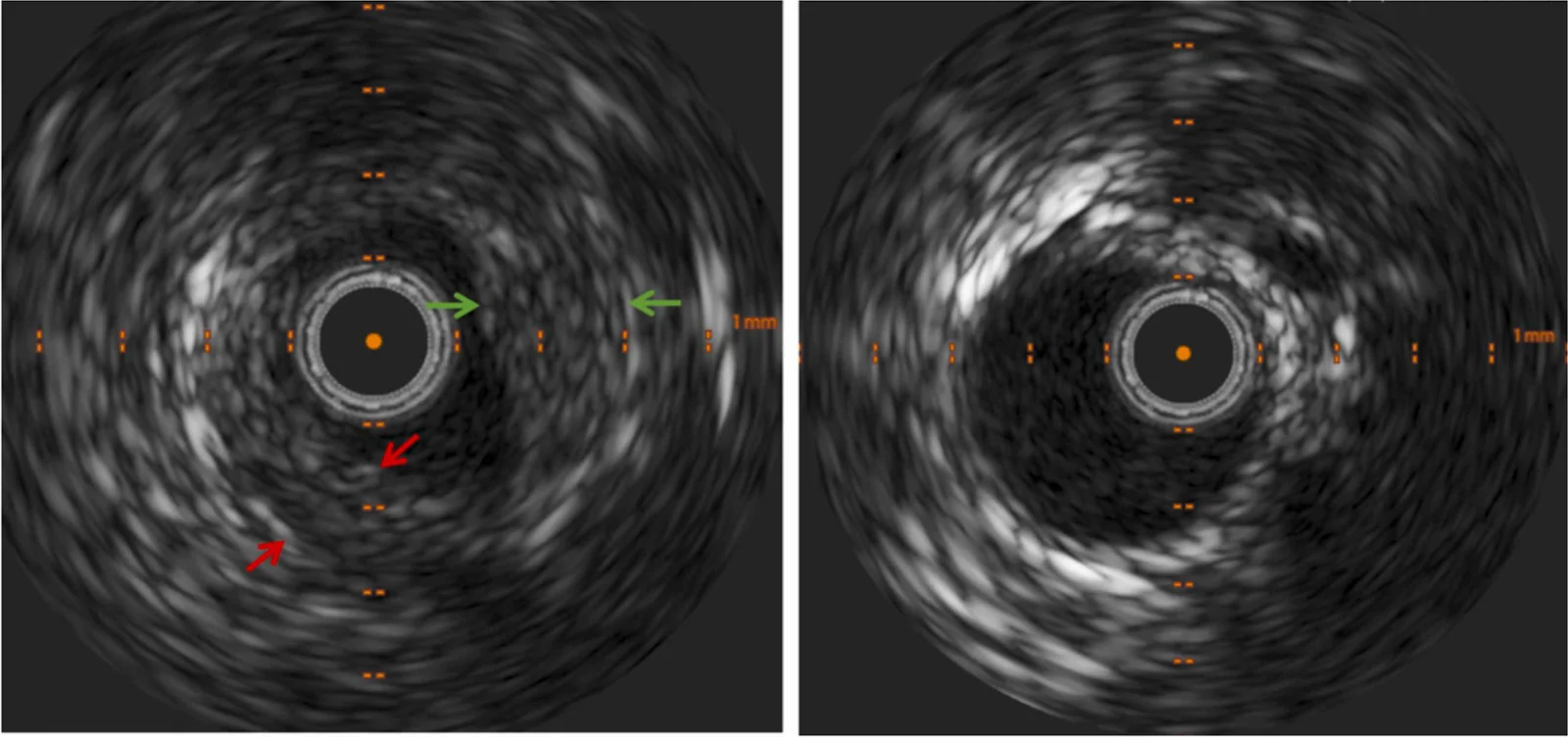
Intravascular ultrasound reveals intraluminal narrowing of the pulmonary vein. Intraluminal thickening (left) is highlighted with red and green arrows, obtained with IVUS 34 months post-transplant. A healthy pulmonary vein (right) is shown for comparison.

A female infant with a history of VACTERL, tracheostomy, PH, and severe PVS requiring multiple interventions underwent a bilateral lung transplantation for progressive disease at 17 months of age. Her immunosuppression regimen included anti-thymocyte globulin, prednisolone, tacrolimus, and azathioprine. Four months after transplant, she underwent cardiac catheterization for bilateral branch pulmonary artery (PA) anastomotic stenosis. The PA anastomoses were dilated, and the pulmonary venous cuff anastomoses were noted to be patent bilaterally (Figure 1A). Eleven months after transplant, she had a chest CT with contrast performed, which demonstrated post-surgical changes and consolidative opacifications in the lower lobes bilaterally, while visualizing the left inferior pulmonary vein (Figure 2A). In the first two years after transplant, she required frequent hospitalizations for respiratory infections without proven acute cellular rejection, and had two echocardiograms (four months and seven months postoperatively) revealing unobstructed pulmonary venous flow. She continued to have recurrent admissions, and roughly 34 months post-transplant, had an echocardiogram revealing a right lower pulmonary vein gradient of 6–7 mmHg, a cardiac CT demonstrating severe left PVS/atresia (Figure 2B), and a perfusion scan indicating asymmetrical lung perfusion (19% left vs 81% right)**.** Cardiac catheterization subsequently demonstrated features of post-capillary pulmonary hypertension (CI: 4.8 L/min/m²; mRPA: 32 / mLPA: 29 mmHg; half-systemic RV pressures; RPCWP: 15 / LPCWP: 18 mmHg). PVR could not be calculated in the setting of multivessel PVS. Angiography revealed severe stenosis, intraluminal narrowing, and wall thickening of the distal left lower vein, with varying degrees of ostial stenosis in the left upper, lingular, and right middle veins, the last with distal hypoplasia (Figure 1B). Intravascular ultrasound (IVUS) of an obstructed vein was performed to help confirm disease recurrence, demonstrating intraluminal thickening without significant signs of external compression (Figure 3, Video 1). Serial balloon dilation was performed with a reduction of RV pressure to less than half-systemic pressures. She was diagnosed with recurrent PVS and placed on sirolimus for medical management, replacing azathioprine. Stent placement was ultimately deferred given the patient's immunocompromised status and her improvement with medical management and balloon dilation.^1^

Supplemental material related to this article can be found online at doi:10.1016/j.jhlto.2026.100650.

The following is the Supplemental material related to this article Video S1.

Video S1

## Discussion

PVS can occur following repair of congenital heart disease, in association with pulmonary diseases, including bronchopulmonary dysplasia, or without an identifiable cause.^2^, ^3^ Disease-modifying therapies are limited, and refractory cases are referred for lung transplantation. Post-transplant PVS occurs in approximately 1.4% of lung transplants and is frequently secondary to technical issues at the anastomosis site.^4^ Because the venous anastomosis is typically performed at the junction of the pulmonary vein and left atrium, proximal involvement is more common, with suture-related narrowing, angulation, and external compression contributing. Most cases are diagnosed in the early postoperative period, with symptoms including hypoxemia, PH, and signs of graft dysfunction.^5^ The recurrence of distal intraluminal/primary PVS after lung transplantation has not been reported. In this case, several findings argue against a venous anastomosis complication. Cardiac catheterization performed four months after transplant demonstrated a patent venous anastomosis without a gradient, along with cross-sectional imaging and echocardiogram, all performed within 12 months of transplantation, showing no venous abnormalities. The hemodynamic data obtained during cardiac catheterization, bilateral distribution of disease, and timing of diagnosis (∼3 years post-transplant) also support disease recurrence. This may have been a result of inflammation from recurrent pulmonary infections, shear stress of the pulmonary vein, or a genetic predisposition. Finally, it remains possible that venous congestion from underlying or developing PVS contributed to her recurrent infections and chest CT changes, especially in the absence of rejection.

## Conclusion

We present the first known case of recurrent PVS diagnosed after lung transplantion in a pediatric patient. This case highlights the need for careful pre-transplant counseling regarding the potential risk of recurrent PVS, the importance of frequent post-transplant surveillance with echocardiography and low threshold for advanced diagnostic testing, and the need for future studies to optimize surgical approach, surveillance protocols, and adjunctive therapies in patients transplanted for PVS.

## Declaration of Competing Interest

The authors declare the following financial interests/personal relationships which may be considered as potential competing interests: Dr. Benden is a past guest editor and current deputy editor for this journal and was not involved in the editorial review or the decision to publish this article. The other authors declare that they have no known competing financial interests or personal relationships that could have appeared to influence the work reported in this paper.
